# Supplementary figures and images for: Assessing the validity of fecal sampling for characterizing variation in threespine stickleback’s gut microbiota
Source: PLoS One. 2023 Sep 21;18(9):e0290875. doi: 10.1371/journal.pone.0290875 (PMC10513271; doi:10.1371/journal.pone.0290875)

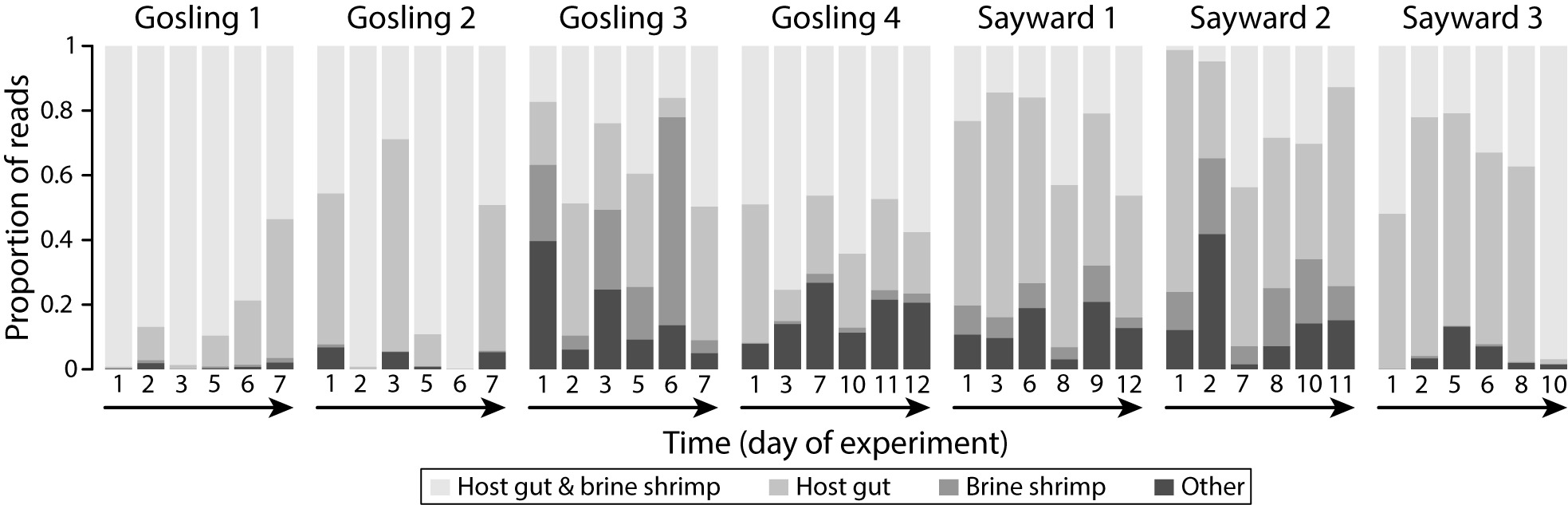

Supplement: S1 Fig — Smaller proportions were shared exclusively with diet (brine shrimp) or with neither the gut microbiota nor diet (other). (TIF) [file pone.0290875.s001.tif]

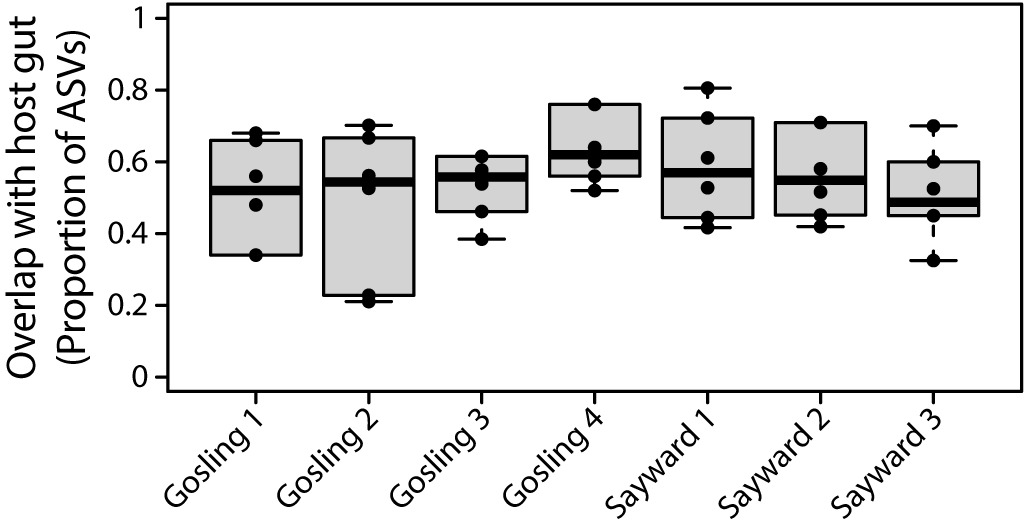

Supplement: S2 Fig — (TIF) [file pone.0290875.s002.tif]

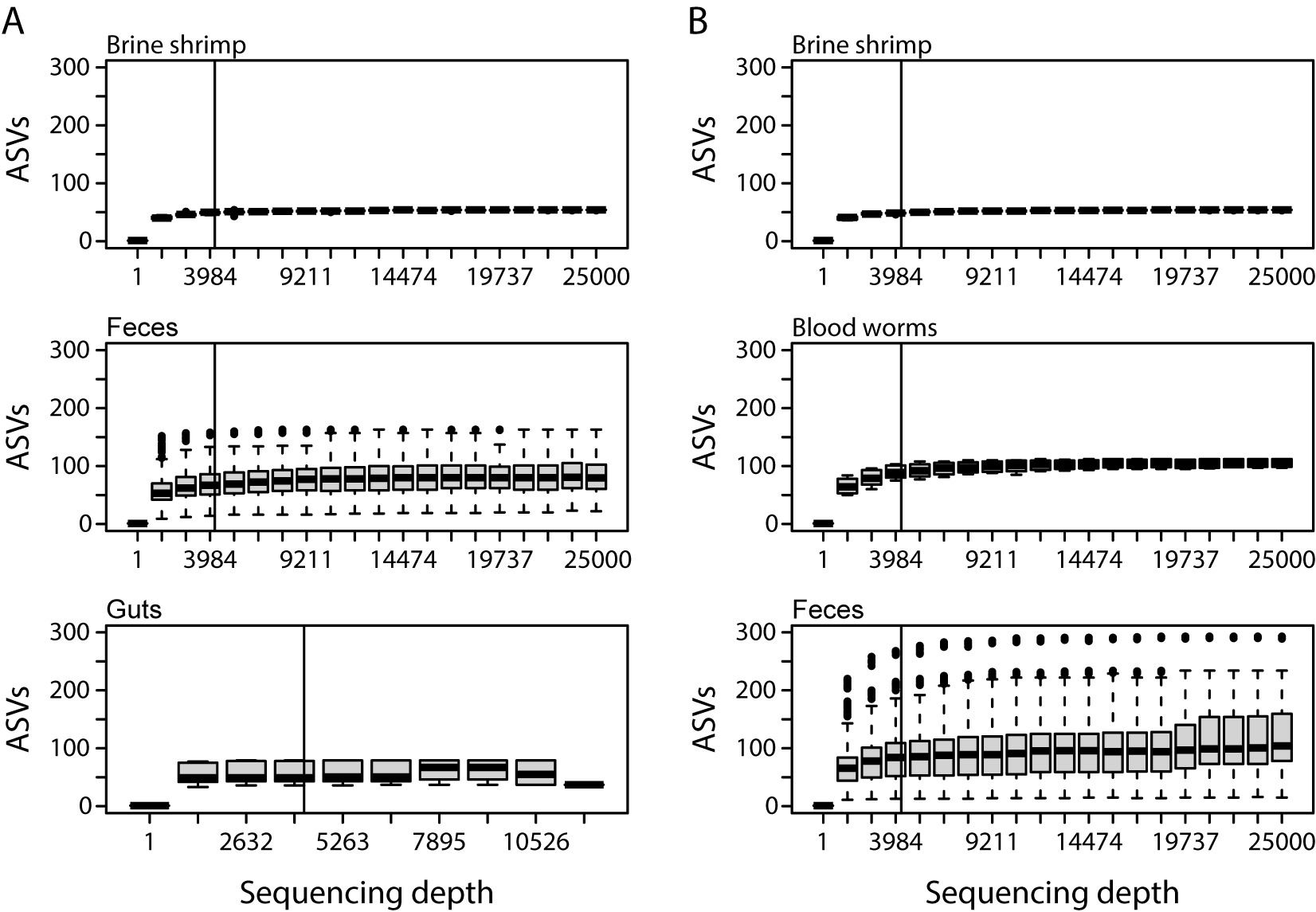

Supplement: S3 Fig — Alpha diversity estimates (ASV richness) at different rarefaction depths for different source materials (feces, guts, diet items) of the first (A) and second (B) experiment. The investigated sequencing depths range from 1 to 25,000 reads. We chose 4238 and 4275 reads as the sequencing depths at which the data was rarefied for the first and second experiment, respectively (indicated by vertical lines). At these sampling depths, a large proportion of the microbial diversity is captured across the different sample types. (TIF) [file pone.0290875.s003.tif]

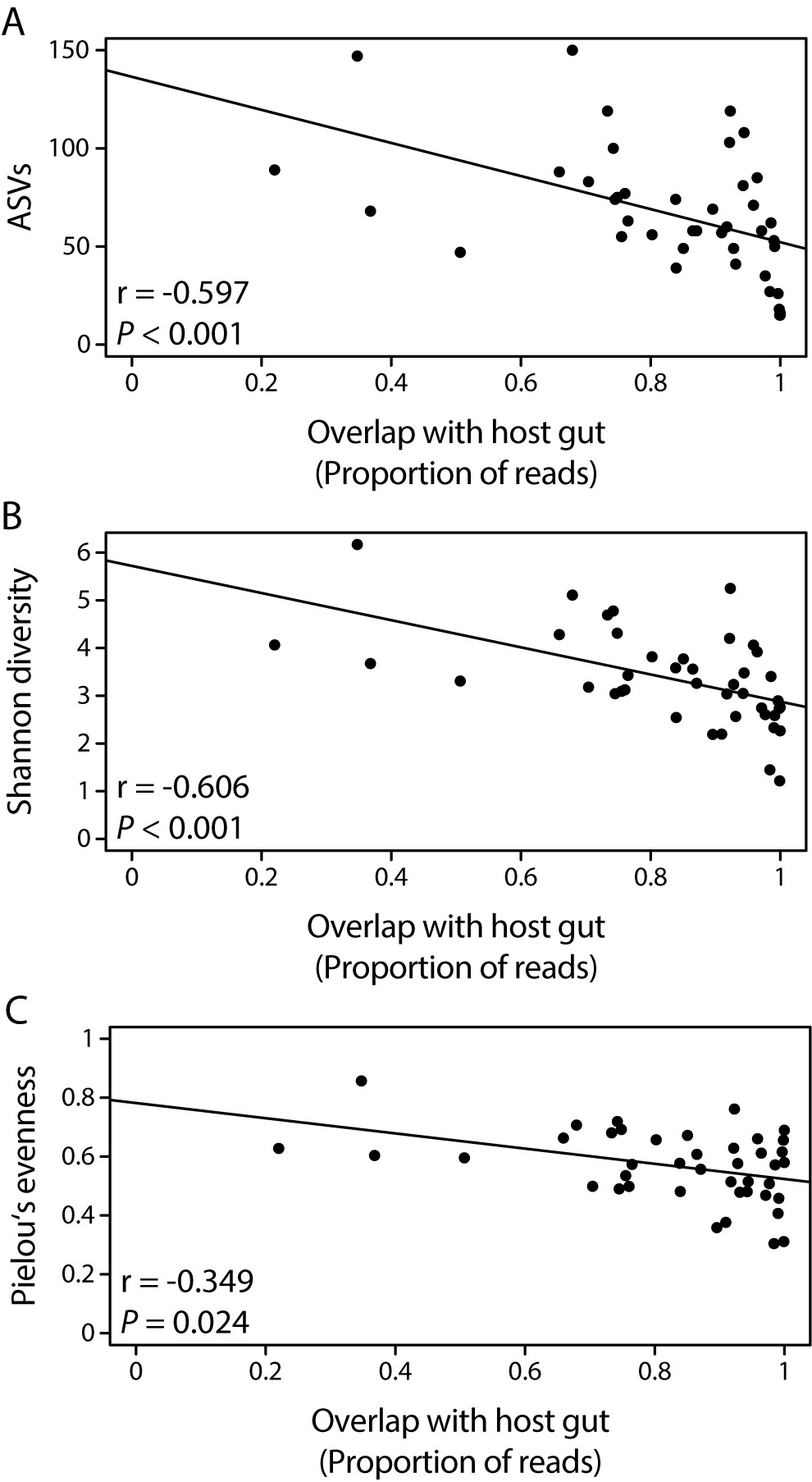

Supplement: S4 Fig — Across fecal samples, all three alpha diversity measures, ASV richness (A), Shannon diversity (B), and Pielou’s evenness (C) were negatively correlated with the overlap between bacterial communities of fecal samples and the gut tissue (based on Spearman’s rank correlation coefficient). (TIF) [file pone.0290875.s004.tif]

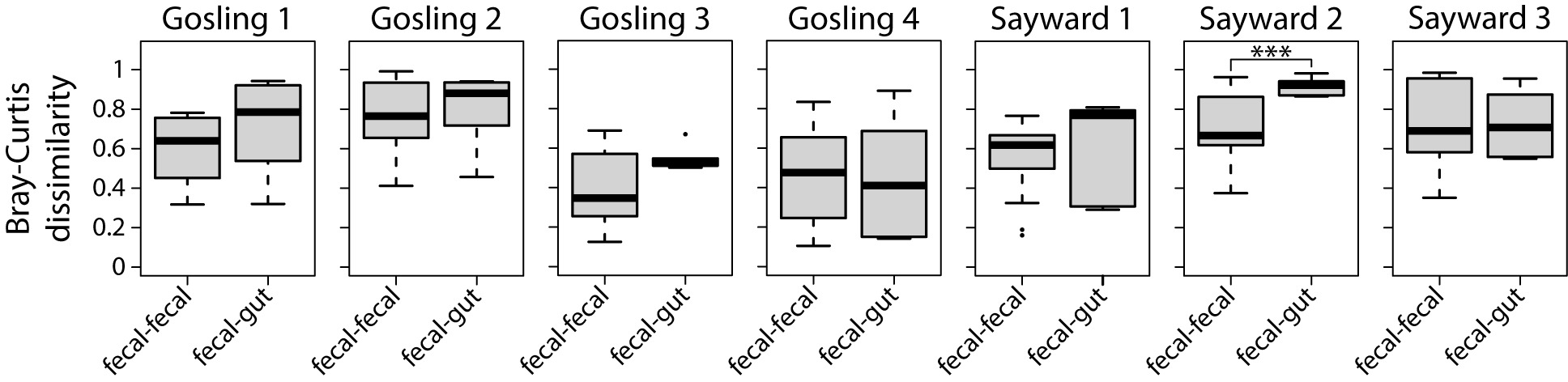

Supplement: S5 Fig — Higher values indicate stronger dissimilarity among samples. Wilcoxon rank-sum tests, ***P < 0.001 (adjusted for multiple comparisons using Bonferroni correction). (TIF) [file pone.0290875.s005.tif]
